# Determinants of piglet gut microbiome colonization: roles of genetics, nutrition, therapeutics, and the impact of enteric pathogens like PEDV

**DOI:** 10.3389/fcimb.2025.1626239

**Published:** 2025-08-25

**Authors:** Yanzhuo Lv, Yu Zhou, Hongde Lu, Hong Dong, Zhiyuan He

**Affiliations:** ^1^ Beijing Key Laboratory of Traditional Chinese Veterinary Medicine, Beijing University of Agriculture, Beijing, China; ^2^ Beijing Engineering Research Center of Chinese Veterinary Medicine, Beijing University of Agriculture, Beijing, China

**Keywords:** porcine epidemic diarrhea virus (PEDV), intestinal microbiota of piglets, herbal preparations, traditional Chinese medicine (TCM), gut microbiota

## Abstract

The gut microbiota of piglets is crucial for intestinal health and immune function, yet highly susceptible to various factors. Multiple factors such as Genetic and Sow Factors, feeding environment, diet and pathogen combine to shape the gut microbiota of piglets. PEDV, a highly pathogenic and transmissible virus, disrupts the gut microbiota by damaging the intestinal epithelial barrier, leading to microbial imbalance, weakened gut immunity, and severe diarrhea. In this review, we systematically investigated the factors affecting microbial colonization in the gastrointestinal tract of piglets and the effects of PEDV infection on intestinal microecology, intestinal epithelial barrier and mucosal immunity. Meanwhile, the unique potential of Chinese herbal medicines compound represented by Qiwen Huangbai San in repairing the barrier, remodeling the flora and enhancing the immunity was discussed in depth. Through the above multidimensional perspectives, this review aims to provide a scientific basis and an effective preventive strategy for the construction of a comprehensive prevention and control program centered on Chinese herbs to alleviate the intestinal damage caused by PEDV in piglets.

## 1 Microbial colonization of the intestinal tract of piglets

The intestinal microbiota of piglets plays a crucial role in their health, particularly in immune system development, digestion and absorption, and disease resistance. During the transition from maternal dependence to independent growth, the gut microbiota undergoes dramatic changes. Studies have shown that gut microbiota interact with intestinal epithelial cells to modulate the function of the immune system, thereby enhancing piglets’ immune response (Zheng et al., 2020). A healthy gut microbiota can strengthen intestinal barrier function, reduce inflammatory responses, and play a significant role in defending against the invasion of foreign pathogens (Guevarra et al., 2019). An appropriate gut microbiota can regulate the immune system, prevent excessive immune responses, and reduce the occurrence of intestinal diseases (Yang and Cong, 2021). During weaning, due to changes in diet and environment, the gut microbiota changes significantly, which can easily lead to intestinal discomfort and diarrhea (Li et al., 2018). Studies have indicated that appropriately adjusting the gut microbiota, such as by adding probiotics or prebiotics, can help piglets better cope with weaning stress, improve intestinal barrier function, and thus reduce the occurrence of diarrhea and other intestinal problems (Wang et al., 2019; Xiong et al., 2019). In addition, theintestinal microbiota of piglets can inhibit pathogen colonization through competition, thereby reducing the risk of diarrhea and other intestinal diseases (Li et al., 2022). Furthermore, the intestinal microbiota of piglets is also crucial for health maintenance and disease prevention. Gut microbiota, through competitive interactions with pathogens, can not only inhibit the growth of harmful pathogens but also activate the immune system of piglets, enhancing their disease resistance (Choudhury et al., 2021). Probiotics such as *Lactobacillius* and *Bifidobacterium* can reduce the colonization of harmful pathogens by competing for adhesion sites and secreting antimicrobial substances, thereby reducing the occurrence of intestinal diseases in piglets (Pang et al., 2022). At the same time, gut microbiota regulates the pH of the intestine through metabolic products, further inhibiting the growth of pathogens and enhancing the disease resistance of piglets (Beaumont et al., 2021). The homeostasis of the gut microbiota is not only directly involved in the development of the immune system and the enhancement of disease resistance but also affects the growth and development of piglets by regulating digestive and absorptive functions. The interaction between gut microbiota and intestinal epithelial cells is crucial for regulating the function of the immune system, helping piglets to enhance their immune response to pathogens (Kayama et al., 2020). A healthy gut microbiota strengthens the intestinal barrier function, reduces inflammatory responses, and plays a significant role in defending against the invasion of foreign pathogens (Ghosh et al., 2021; Yang and Cong, 2021). An appropriate gut microbiota can regulate the immune system, prevent excessive immune responses, and reduce the occurrence of intestinal diseases. Therefore, maintaining the balance of the gut microbiota is irreplaceably important for the health maintenance and disease prevention of piglets.

Microorganisms in the environment enter the piglets after birth, and a portion of the flora colonizes the intestinal tract. For example, microbes enter the body, consuming colostrum, and selecting microbes that use nutrients in breast milk as substrates to initially colonize and then survive and reproduce (Jiang et al., 2019). Microbial diversity increases with host age, gradually forming a stable microbiota that plays an important role in host growth and health. It is widely believed that the colonization of the piglet gut microbiota begins at birth, but there remains controversy over whether microbial colonization occurs during the fetal period. Over the past five years, studies have been conducted to isolate or sequence viable bacteria and bacterial DNA (e.g., Lactobacillus, Proteus, and Firmicutes) from amniotic fluid, placenta, and fetal stools at 70–110 d of gestation, suggesting that microorganisms may be pre-seeded via the placenta-amniotic cavity pathway (Lim et al., 2023). Although this “intrauterine colonization hypothesis” has been questioned due to the risk of low-biomass contamination and unclear functional significance (Xiao and Zhao, 2023), sterile cesarean section models and multi-omics tracking have preliminarily demonstrated significant overlap between fetal flora signaling and early postnatal intestinal flora. However, aseptic cesarean section models and multi-omics tracking have preliminarily demonstrated that fetal flora signals significantly overlap with early postnatal gut flora, and are highly correlated with sow milk and skin flora, suggesting that vertical transmission across the placenta or through the amniotic fluid-oral cycle should not be ignored. Therefore, whether microbial colonization of piglets begins at birth or partially occurs during the fetal period remains one of the central controversies in current research on porcine microecology and perinatal immunity.

The colonization sequence of piglet intestinal microbiota is aerobic bacteria, followed by facultative anaerobes, and obligate Anaerobes (Bian et al., 2016) The development of the piglet gut microbiota exhibits stage-like and dynamic changes. In the first few days after birth, the gut is mainly dominated by facultative anaerobes (such as Escherichia coli and Enterobacteriaceae) (Butkovich et al., 2025). Gut microbiota colonization can be divided into three stages: the initial stage, the transition stage, and the stable stage. The initial stage is from birth to one week of age, mainly composed of maternal microbes. Sows transmit microbes through childbirth, nursing, and contact, playing a key role in the early establishment of the gut microbiota and the maturation of the barrier (Nowland et al., 2022). Oligosaccharides and Antimicrobial peptides in milk promote the proliferation of beneficial bacteria such as Lactobacillius and Bifidobacterium and inhibit the colonization of potential pathogens (Ferret-Bernard et al., 2020). The transition stage from one to four weeks of age in piglets, with an increase in microbial diversity, Lactobacilli and Bifidobacteria becoming the main bacterial community, the gut environment tends to stabilize, and breast milk and solid food begin to jointly influence the composition of the microbiota (Saladrigas-García et al., 2021). The stable stage is after four weeks of age in piglets, with the microbiota structure significantly impact and showing individual specificity (Butkovich et al., 2025). Bacteroides, Clostridium, and Lactobacillus play an important role in maintaining intestinal ecological balance, but environmental and dietary changes still have a significant impact on the microbiota (Saladrigas-García et al., 2021). In addition, there are significant differences among in the composition and function of the microbiota in different parts of the piglet intestinal tract. For example, the small intestine is mainly dominated by the Lactobacillus genus, which plays an important role in lactose fermentation and lactic acid production; while in the colon and cecum, Bacteroides and Clostridium ferment dietary fiber to produce Short-chain fatty acids (SCFAs), providing energy for the host and regulating immune responses (Liu et al., 2024; Sun F. et al., 2024). The small intestine, cecum, and colon are key areas for intestinal microbial colonization, each bearing distinct digestive, immune, and metabolic functions. The colonization of different intestinal segments in piglets reflects the significant differences in the microenvironment and physiological functions of different parts of the intestinal tract, playing an important role in digestion, immunity, and metabolism.

**Figure 1:**
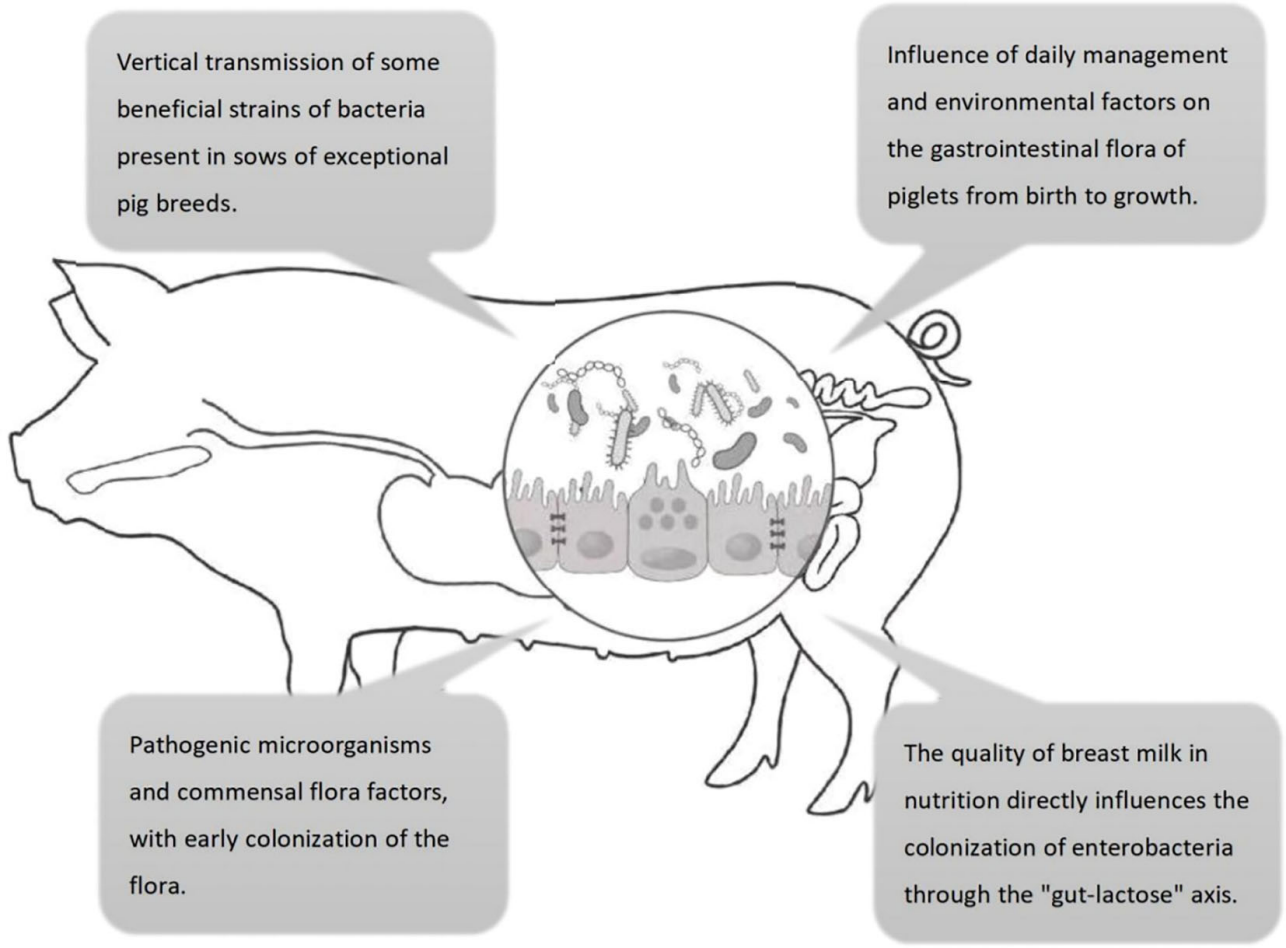
Four key factors influencing the colonization of intestinal flora in pigs. ① maternal vertical transmission; ② environmental husbandry; ③ milk-mediated regulation via the “gut-lactose axis”; ④ pathogen–commensal equilibrium.

The colonization process of intestinal microbiota in piglets plays a crucial role in their health, development of the immune system, and growth performance. Recent studies have indicated that piglet intestinal microbiota colonization is influenced by a variety of factors, including maternal microbiota transmission, mode of delivery, diet, antibiotic use, environmental factors, weaning stress management, and genetic background. These factors interact through multiple mechanisms to affect the establishment of the intestinal microbiota, thereby influencing piglet intestinal health and immune function (**Figure 1**).

### 1.1 Genetic and sow factors

The porcine intestinal microbiota is shaped not only by external factors such as housing environment and feed composition but also significantly influenced by the host’s genetic background. Research has demonstrated marked differences in gut microbial composition among different pig breeds, which may be attributed to host genetic effects on microbial colonization, metabolic functions, and immune regulation. For instance, a 16S rRNA high-throughput sequencing study comparing the fecal microbiota of four boar breeds—Duroc, Yorkshire, Landrace, and Hampshire—identified 783 shared operational taxonomic units (OTUs), alongside breed-specific microbial communities. *Firmicutes* and *Bacteroidetes* were the dominant phyla, with *Clostridium*, *Bacillius*, and *Bacteroidies* being the predominant classes. Most of the dominant genera were capable of producing SCFAs. Notably, breed-specific differences in volatile fatty acid levels were observed: Landrace pigs exhibited the highest butyrate levels, whereas Hampshire pigs had the highest acetate and propionate concentrations. Moreover, Hampshire pigs displayed a higher Shannon diversity index, while Landrace pigs showed a lower ACE richness index, suggesting that genetic background significantly influences both the structure and function of the gut microbiota (Xiao et al., 2017). In another study, Duroc and Yorkshire boars were systematically compared at a body weight of 100 kg for growth performance and gut microbial composition. Microbial analysis revealed that Yorkshire boars had significantly higher ɑ-diversity compared to Duroc boars, with distinct microbial community structures between the two breeds. Specifically, variations in the relative abundances of *Bacteroidetes*, *Prevotella*, and *Ruminococcus* were potentially associated with growth performance and lean meat percentage in Yorkshire pigs. Functional prediction analyses further indicated significant differences in microbial metabolic pathways between the two breeds, implying a potential role of the gut microbiota in mediating breed-specific differences in growth performance (Du et al., 2023). Additionally, studies have shown that wild boars harbor a significantly higher abundance of *Actinomycetes* in their intestines compared to domestic pigs, which are predominantly colonized by *Bacteroidetes*. Functionally, the gut microbiota of wild boars exhibits enhanced pathogen resistance and crude fiber digestion capabilities, possibly due to the production of antimicrobial and immunomodulatory compounds by *Actinobacteria*. In contrast, domestic pigs show a higher abundance of *Lactobacillus*, which may be linked to intensive farming practices and antibiotic usage (Yang et al., 2020). Collectively, these findings highlight the diversity and functional differences in the porcine gut microbiota across various genetic backgrounds and breeds, providing critical insights into swine health, growth performance, and nutritional metabolism.

The intestinal flora of the sow plays a crucial role in the colonization of the intestinal flora of the piglet. The sow’s health status, dietary habits, and immune system function during gestation affect the composition of intestinal microbial community, which is passed on to her piglets through the birthing process forming the intestinal microbiota of the piglets in early life (Guevarra et al., 2019). Specifically, piglets acquire their maternal microbiota during birth through exposure to bacteria in the vagina, udder and breast milk of the sow. This process provides the basis for pigletintestinal microbiota colonization and has a profound effect on piglet health (Guevarra et al., 2019). However, the sow’s health status and factors such as her diet and antibiotic use may also influence the composition of the maternally-derived flora. For example, antibiotic use by sows during pregnancy may lead to an imbalance in their intestinal flora, thereby reducing the delivery of beneficial flora to piglets. Therefore, healthy management of sows and rational antibiotic use are essential for establishing piglet intestinal flora.

According to relevant studies, sows play a significant role in the colonization of piglet gut microbiota. In the early stages after birth, sows vertically transmit their own microorganisms to piglets. These microorganisms mainly include beneficial bacterial phyla such as *Firmicutes*, *Bacteroidota*, and *Proteobacteria* (Lim et al., 2023). Research has found that within 7 days after birth, the sow’s contribution to the piglet gut microbiota is as high as 31.68%. Although this proportion gradually decreases over time, it remains at 13.33% even 10 days after weaning (Tancredi et al., 2025). The microbial species transmitted by sows to piglets are diverse, including beneficial bacteria such as Lactobacillus acidophilus with probiotic activity and SCFAs producers, as well as some potential pathogens like *Clostridium sensu stricto1* and *Escherichia-Shigella*. These microbial species can stably colonize in the piglet gut and play important roles during the early development stage. Additionally, the ɑ-diversity of piglet gut microbiota increases with age and tends to stabilize after weaning. Factors such as individual differences among sows, piglet age, and pen environment after weaning all have significant effects on microbial diversity. After weaning, the microbial exchange among pen mates contributes to 53.54% of the piglet gut microbiota, significantly reshaping the microbial composition (Tancredi et al., 2025). These microorganisms transmitted from sows can stably colonize in the piglet gut and persist until after weaning, providing an important foundation for piglet gut health and immune system development.

### 1.2 Daily management and environmental factors

Daily management and environmental factors have a multifaceted impact on the intestinal microbiota of piglets, shaping the structure and function of the gut microbiota from birth to growth. Environmental conditions play a significant role in the colonization of the intestinal microbiota in piglets. Newborn piglets quickly transition from a relatively sterile environment to one where they are exposed to a variety of microbes. During this process, microbes from the sow’s birth canal, feces, and the surrounding environment become the initial colonizers in the piglet’s gut (Chen et al., 2022). Studies have shown that environmental pollution, inappropriate temperature and humidity, and the presence of pathogenic microorganisms in the air can all affect the intestinal health of piglets (Saha et al., 2024). Piglets are more susceptible to pathogenic infections in unclean or overly humid environments, leading to an imbalance in the gut microbiota and an increased incidence of diarrhea and other intestinal diseases (Chen et al., 2022). Conversely, a healthy and clean breeding environment helps maintain the diversity of the intestinal microbiota, enhancing the immunity and growth performance of piglets (Quan et al., 2023). Research has found that the gut microbiota structure of pigs from the same litter is highly similar, not only because they share the same genetic background but also due to their identical living environments. Under different breeding conditions, the gut microbiota of piglets shows significant differences. For example, piglets raised under low hygiene conditions with sows have a more diverse gut microbiota, with a significant increase in beneficial bacteria such as the *Lactobacillus genus* and a significant decrease in conditional pathogens (Mei et al., 2025).

Daily management practices, particularly weaning, have a significant impact on the gut microbiota of piglets. Weaning is a sudden process that leads to rapid ecological succession in the piglet’s gut microbiota, a shift known as microbial translocation (Plaza-Diaz et al., 2014). During weaning, piglets transition from milk to plant-based solid feed, and this sudden change in diet is a significant factor affecting the structure of the microbial community (Han et al., 2024). Studies indicate that after weaning, microbial diversity in piglet guts increases, while variability due to individual differences decreases, and gut bacterial diversity continues to rise as piglets grow older.

### 1.3 Pathogenic microorganisms and symbiotic flora factors

Pathogenic and symbiotic microorganisms exert multifaceted influences on piglet intestinal microbiota, playing a pivotal role in their health and disease susceptibility. Pathogens, such as *Enterotoxigenic Escherichia Coli* (ETEC), *Salmonella*, and *Clostridium*, are the main bacterial pathogens causing diarrhea in piglets (Wu et al., 2020). These pathogens can affect the gut barrier function of piglets through adhesins and toxins, leading to inflammation and diarrhea. In addition, ETEC can cause diarrhea in neonatal and weaned piglets by producing heat-stable and heat-labile enterotoxins (Jin et al., 2024).

Beyond pathogenic microorganisms, commensal microbiota also shapes the colonization of piglet gut microbiota. Symbiotic flora, such as *Lactobacillus* and *Bifidobacterium*, are crucial for the gut health of piglets. They help maintain the integrity and function of the gut barrier by producing SCFAs and other metabolic products (Connolly et al., 2024; Mann et al., 2024). Butyrate and other SCFAs from commensal microbiota help stabilize intestinal pH in piglets, promoting microbial colonization (Su et al., 2022).Not only that, but SCFAs can also provide energy for intestinal epithelial cells, stimulate the expression of their receptors, activate downstream pathways, and thus improve intestinal development (Su et al., 2022). Additionally, the supplementation of probiotics can improve the richness of the gut microbial community and shape a gut microbiota oriented towards beneficial bacteria, resisting the infection of pathogenic microorganisms (Frese et al., 2015). For example, *Lactobacillus* can prevent pathogens from attaching to the mucosal surface through competitive exclusion mechanisms and secrete antimicrobial substances, such as Bacteriocins, Organic acids, and Hydrogen peroxide (Haghshenas et al., 2023). These antimicrobial substances have a direct antibacterial effect on competitive pathogens and can prevent the colonization of pathogens in the piglet’s gut.

### 1.4 Nutritional factors and pharmaceutical factors

The diet of piglets plays a central role in the colonization of the intestinal microbiota. Shortly after birth, piglets are rapidly colonized by complex microbial communities, the importance of which to the host’s health is becoming increasingly evident. During the lactation period, the milk of sows provides piglets with an important selective advantage, allowing certain microbes to dominate in the gut ecosystem (Reyman et al., 2022). This milk-shaped microbial community is referred to as the “Milk-Oriented Microbiome” (MOM), which exerts a controlling effect on the gut microbiome of mammals (Natal et al., 2024). In addition to rich nutrients, the milk is abundant in probiotics such as *Lactobacillus* and *Bifidobacterium*, which help establish a healthy gut microbiota structure (Xing et al., 2024). Furthermore, immunoglobulins in the milk have a positive impact on the development of the piglets’ immune systems (Connolly et al., 2024). However, the abrupt dietary transition of weaning causes a shift in the intestinal microbiota of piglets. Post-weaning, the microbial community is functionally distinct from that during the nursing period, particularly in the degradation pathways of Plant Glycosides (Gresse et al., 2017).

In addition to this, the use of antibiotics is one of the significant factors affecting the intestinal microbiota of piglets. Studies have found that antibiotics can effectively kill pathogens, suppress the growth of harmful bacteria, temporarily improve the structure of the intestinal microbiota, and reduce the occurrence of some intestinal diseases (Dong et al., 2021). However, this non-selective bactericidal effect also destroys the symbiotic bacteria in the gut. It can lead to an imbalance in the intestinal microbiota, promote the colonization of harmful pathogens, reduce the number of beneficial bacteria in the gut, and even lead to the emergence of drug-resistant strains, which poses a potential threat to the immune system and intestinal function of piglets (Bai et al., 2021). In contrast, traditional Chinese medicine (TCM) has shown unique advantages in regulating the gut microbiota. The mechanism of action of TCM mainly focuses on adjusting the overall balance of the gut microbiota to suppress the growth of pathogens, rather than directly killing them (Ye et al., 2022). This approach is more in line with the principle of “yin-yang balance” in nature, that is, by enhancing the vitality of beneficial bacteria in the gut to suppress the overgrowth of harmful bacteria, thus maintaining gut health (Tan et al., 2019). Various active components in TCM, such as Polyphenols, Alkaloids, and Volatile oils, have been proven to have a positive impact on the gut microbiota, including promoting the growth of beneficial bacteria, inhibiting the adhesion and invasion of pathogens, and regulating intestinal immune function (Tan et al., 2019).

In the study by Guang Chen, weaned piglets were fed with fermented traditional Chinese medicine (TCM) after weaning, and the results showed significant improvements in growth performance and gut health (Chen G, et al., 2023). Compared with the control group (CON), piglets in the LFHM and HFHM groups, which were supplemented with fermented TCM, had significantly increased final weight (FW), average daily feed intake (ADFI), and average daily gain (ADG) (P<0.01). The apparent digestibility of crude protein (CP) was significantly improved (P<0.05), and the activities of trypsin, ɑ-amylase, and lipase were significantly enhanced (P<0.01). In terms of gut health, the villus height (VH) in the jejunum of piglets in the LFHM and HFHM groups increased, and the crypt depth (CD) also increased. The concentration of isovalerate in the HFHM group was higher than that in the CON and LFHM groups (P<0.05), while the concentration of Butyrate was lower than that in the CON and LFHM groups (P<0.05). Additionally, 16S rRNA sequencing results showed that the LFHM and HFHM groups significantly affected the microbial α-diversity indices in the colon of weaned piglets (P<0.01) and increased the relative abundance of beneficial bacteria (such as *Lactobacillius*). These results indicate that fermented TCM can significantly improve the growth performance of weaned piglets by promoting the secretion of intestinal digestive enzymes, altering gut microbial diversity, regulating the content of short-chain fatty acids in the gut, promoting gut health, and enhancing nutrient digestibility. In addition, the study found that piglets in the 0.05% and 0.2% Guizhi Lizhong (GLZ) supplemented groups performed better than those in the control and antibiotic groups in terms of growth performance, diarrhea rate, and gut damage (P<0.05). High-throughput sequencing analysis revealed that GLZ treatment significantly increased the abundance of beneficial bacteria, while antibiotic treatment decreased beneficial bacteria. This successfully demonstrated that Guizhi Lizhong Decoction can be used as an alternative to antibiotics in feed, not only effectively reducing gut damage but also improving gut microbiota, thereby promoting the growth of weaned piglets (Wang et al., 2024). In summary, the gut microbiota of weaned piglets are prone to imbalance under the influence of dietary changes and antibiotic use. TCM, through multiple mechanisms such as regulating gut microbiota, enhancing the vitality of beneficial bacteria, promoting gut health, and improving nutrient digestibility, has shown great potential as an alternative to antibiotics, providing new ideas and methods for ensuring the healthy growth of piglets.

## 2 Effect of PEDV on the intestinal flora of piglets

Porcine epidemic diarrhea (PED) is an acute or subacute swine intestinal disease caused by the porcine epidemic diarrhea virus (PEDV), characterized clinically by sudden diarrhea and vomiting. Pigs of all age groups are susceptible, with suckling piglets being the most vulnerable, having a mortality rate as high as 80% to 100%. At the end of the 20th century, PEDV was first introduced into China, exhibiting regional and seasonal outbreak characteristics (Lee, 2015). During its transmission, the virus mutated from the initial GI classical strain to the highly pathogenic GII strain and continued to evolve. The impact of PEDV variant strains is not limited to China; since 2010, they have triggered outbreaks worldwide, including in Japan, South Korea, Thailand, Canada, and Mexico, causing significant economic losses to the global swine industry (Shamsi et al., 2022).

PEDV infection often leads to massive shedding and functional disruption of intestinal villous epithelial cells, thereby impairing digestive and absorptive functions (Ducatelle et al., 1982). Vomiting and decreased appetite, common complications of PED, exacerbate dehydration. The induction of vomiting by serotonin and the increase in pro-inflammatory cytokine responses are partly responsible for the decreased appetite (Jung and Saif, 2015). In addition, piglets infected with PEDV often suffer from hyperkalemia and acidosis due to the loss of bicarbonate, and these metabolic disorders further affect cardiac function.

**Figure 2:**
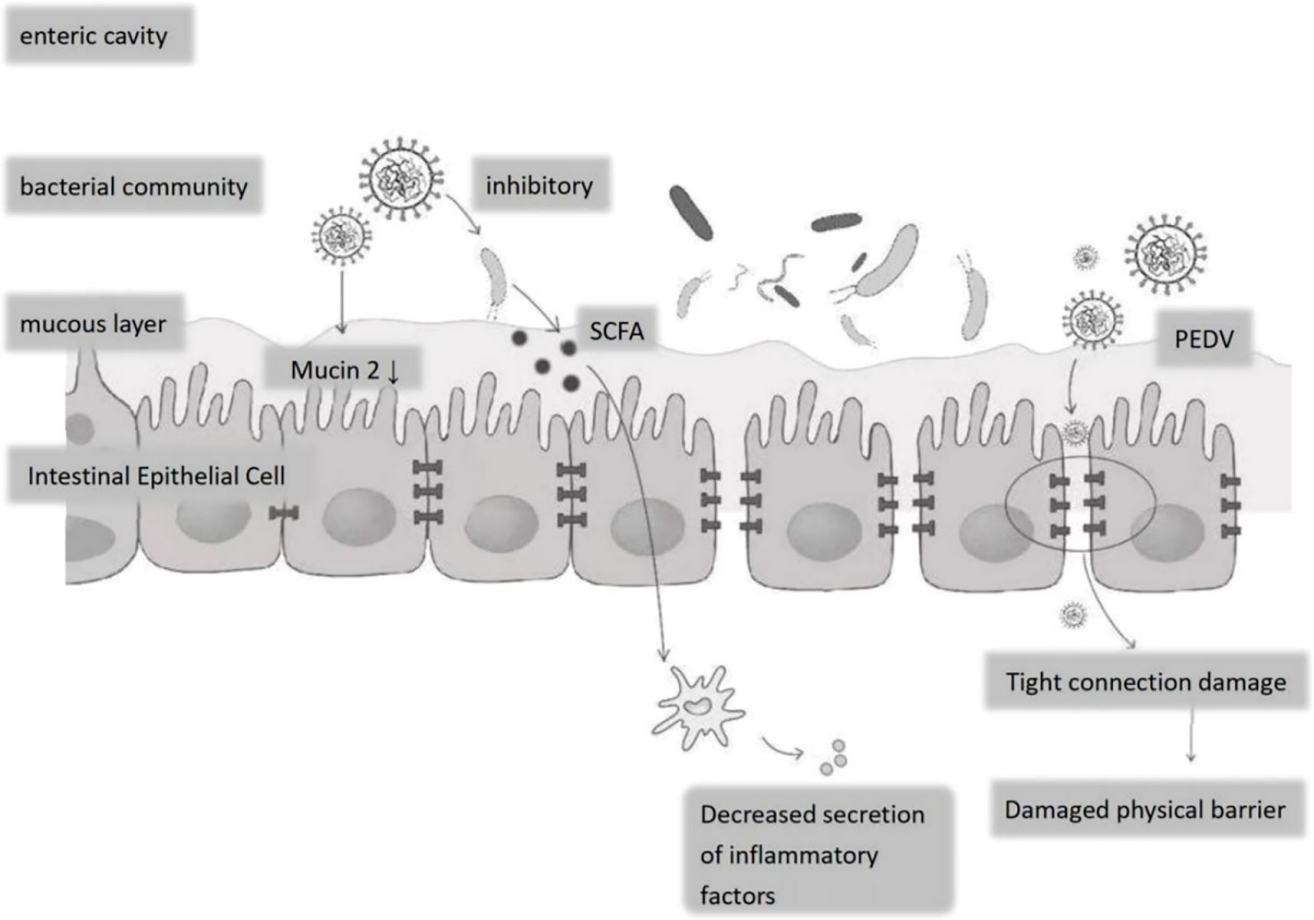
Effects of PEDV on the epithelial structure and bacterial flora of porcine small intestine. Following PEDV infection: decreased Mucin-2 secretion by goblet cells thins the mucous layer; tight-junction disruption compromises the epithelial physical barrier; elevated inflammatory cytokine release disrupts the luminal bacterial community, suppressing commensals and allowing expansion of potential pathogens.

The impact of PEDV on the gut microbiota of piglets is a complex and multidimensional process, involving the composition of the intestinal microbiota, intestinal barrier function, and the host’s immune response. Infection with PEDV leads to disturbances in the gut microbiota, which involve changes in both the composition and function of the gut microbiota, thereby disrupting the physical and chemical barriers of the intestine and affecting the immune function of the intestinal mucosa (Song et al., 2017) (**Figure 2**).

### 2.1 Disruption of the physical and chemical barrier of the intestinal tract

Porcine Epidemic Diarrhea Virus (PEDV) infection significantly disrupts the intestinal microbiota of nursing piglets and compromises the physical and chemical barriers of the intestine. Studies have indicated that PEDV infection can lead to alterations in the structure of the gut microbiota, particularly an increase in the number of bacteria associated with diarrhea (Tan et al., 2019). In nursing piglets infected with PEDV, there is an increase in the quantity of pathogenic bacteria in the intestine, including those related to diarrhea such as *Fusobacterium* (ranging from 26.71% to 33.91% in the infected group), whereas in the healthy group, these percentages are 17.85% and 9.88%, respectively (Tan et al., 2019). The proportion of *Proteobacteria* is relatively high, while *Bacteroidetes* is lower in the infected group, suggesting that PEDV infection may alter the balance between beneficial and harmful bacteria in the gut (Tan et al., 2019). The disruption of the gut microbiota affects not only the composition of the intestinal microbes but also the physical barrier of the intestine. The intestinal epithelial cell layer is an essential component of the intestinal physical barrier, and PEDV infection can cause damage to the intestinal epithelial cells, thereby increasing intestinal permeability. This increased permeability may facilitate the invasion of pathogens and the occurrence of inflammatory responses (Liu et al., 2015).

Regarding the chemical barrier, the gut microbiota maintains intestinal health by producing metabolic products such as SCFAs. However, PEDV infection may affect the production of SCFAs, thereby impacting the function of the intestinal chemical barrier (Ali et al., 2020). Specifically, PEDV infection can reduce the number of bacteria that produce SCFAs in the intestine, such as Bacteroides and Clostridium bothrium. The decrease in these bacteria may lead to a drop in SCFAs levels, thus affecting the integrity of the intestinal barrier (Abdelhalim, 2024).

### 2.2 Impact on intestinal mucosal immunity

The intestinal barrier is a crucial component of the mucosal immune system, consisting of various epithelial cells and an underlying layer of immune cells (Lacob and Iacob, 2019). PEDV infection disrupts this barrier, potentially increasing intestinal permeability and susceptibility to secondary infections (Wang et al., 2017). Histopathological lesions caused by PEDV infection in piglets primarily affect the duodenum, jejunum, and ileum, manifesting as villous atrophy, dissolution, glandular damage, and partial epithelial cell shedding. These changes reduce the absorptive surface area of the intestine, impair nutrient absorption, and increase intestinal permeability, facilitating the translocation of pathogens and harmful substances across the barrier (Jung and Saif, 2017).

Studies have demonstrated that PEDV infection significantly reduces the number of goblet cells responsible for mucus secretion and maintaining intestinal integrity (Xiao et al., 2021). The reduction in goblet cells compromises the mucus layer, which is vital for protecting the intestinal epithelium from pathogen invasion (Moran et al., 2012). Furthermore, PEDV infection has been associated with decreased expression of mucin 2, a key component of the mucus layer, further weakening the intestinal barrier (Moran et al., 2012).

Beyond the direct effects on the mucus layer, PEDV infection also impacts immune cells in the lamina propria. The infection activates pro-inflammatory pathways, leading to the release of cytokines such as IL-1β and IL-18, which are associated with a programmed cell death mechanism called Pyroptosis (Moran et al., 2012). Pyroptosis damages the intestinal barrier by creating pores in the cells and releasing inflammatory cytokines, thereby increasing piglets’ susceptibility to PEDV (Zhang et al., 2022).

The integrity of the intestinal barrier is maintained by various tight junction proteins. Research indicates that PEDV infection negatively affects the expression of these proteins, increasing permeability and facilitating viral entry (Zhang et al., 2022). PEDV infection also significantly decreases D-xylose levels and increases Diamine oxidase (DAO) activity, indicating intestinal dysfunction. A reduction in D-xylose, a key marker of intestinal barrier function, reflects compromised barrier integrity, while increased DAO activity suggests mucosal damage and declining barrier function (Benjamin et al., 2000).

Additionally, PEDV infection reduces the activity of Antioxidant enzymes in serum and intestinal tissue while increasing Malondialdehyde (MDA) levels, indicating heightened oxidative stress and resultant oxidative damage (Liu et al., 2020). MDA levels, as a marker of oxidative stress, can assess the extent of cellular damage (Sun et al., 2023). The infection may also impair Glucagon-like peptide-2 (GLP-2) function, a hormone that promotes intestinal growth and enhances mucosal barrier repair (Petersen et al., 2003). Silencing the GLP-2 gene has been shown to increase PEDV replication, suggesting that barrier damage exacerbates PEDV infection (Zhou et al., 2021).

The role of the gut microbiota in regulating PEDV infection is significant. PEDV infection disrupts the microbial balance in jejunal contents and mucosa (Wu et al., 2024). In uninfected piglets, *Firmicutes* are predominant, while *Proteobacteria* are less abundant. Following PEDV infection, this balance shifts, causing significant changes in microbial composition (Tan et al., 2019). Analysis of jejunal samples through 16S rRNA sequencing reveals distinct microbial community differences between infected and uninfected groups (Xing et al., 2024). This indicates that PEDV infection substantially impacts the gut microbiota composition in piglets.

Research has shown that microbial-derived Lithocholic acid (LCA) mediates protective effects against PEDV in piglets. PEDV infection alters the gut microbiota significantly, particularly in Landrace piglets, which lose resistance quickly after infection. However, fecal microbiota transplantation from Min pigs to Landrace pigs alleviated the infection. The study also identified *Lactobacillus reuteri* and *Lactobacillus amylovorus* as critical contributors to resistance, with LCA being significantly associated with these strains and exerting protective functions in animal models (Yu et al., 2011).

Further studies have revealed a potential link between PEDV infection and the gut microbiota. Regulation of the gut microbiota can reduce the incidence and mortality associated with PEDV. For example, traditional Chinese medicine formulations like Pulsatilla decoction have been shown to increase beneficial gut bacteria and suppress harmful populations in PEDV-infected piglets. This aligns with findings that Pulsatilla saponins regulate the gut microbiota composition and alleviate inflammation in rat models (Bárcenas-Preciado and Mata-Haro, 2024).

The gut microbiota also influences intestinal barrier function by producing SCFAs, which are critical for maintaining barrier integrity. SCFAs have been shown to suppress inflammation and promote mucosal repair. SCFAs, especially butyrate, protect the integrity of the intestinal barrier in piglets by transcriptionally up-regulating tight junction proteins (ZO-1, Occludin, Claudin-1) and by supplying approximately 60-70% of colonic epithelial ATP via the TCA cycle, thereby accelerating cell proliferation and repair (Liu, H et al., 2024a). Meanwhile, butyrate inhibits HDAC activity, inhibits IL-6, TNF-α, and IFN-γ, and elevates IL-10/TGF-β; interacts with GPR43/GPR109A on dendritic cells and macrophages to skew the differentiation toward regulatory T cells; and drives the production of IL-22 through the AhR-HIF-1α axis to stimulate the production of antimicrobial peptide (REGIII).γ) and mucin (MUC2) to strengthen the mucosal barrier against the intestinal pathogen PEDV (Liu, H et al., 2024b; Connolly et al., 2024). The reduction in SCFA-producing bacteria following PEDV infection suggests that these molecules may play a protective role in preventing PEDV-induced intestinal damage (Jung et al., 2015; Xing et al., 2024). Thus, modulating the gut microbiota to enhance SCFAs production could help prevent or mitigate PEDV infections (Liu et al., 2015).

### 2.3 Antiviral effects of symbiotic bacteria

We have discovered that various probiotics positively affect the host’s immune response and gut health through different pathways. Research has found that *Bacillus licheniformis HD173* can improve the growth performance and health status of nursery pigs by reducing the levels of pro-inflammatory cytokines IL-1β and TNF-α in serum, while increasing the levels of IL-10 and Superoxide Dismutase (SOD), demonstrating its anti-inflammatory and antioxidant capabilities (Li et al., 2024). This strain can also reduce the number of pathogenic bacteria in the gut, such as *Mycoplasma*, *Vibrio*, and *Vibrio metschnikovii*, and increase the abundance of Butyrate-producing bacteria like *Oscillospira*, *Coprococcus*, and *Roseburia faecis*, thereby enhancing Butyrate production (Li et al., 2024). In addition, *Lactobacillus plantarum PFM 105* has shown probiotic effects in weaned pigs, improving the development of small intestinal villi and promoting growth, while reducing the incidence of diarrhea (Wang et al., 2019). Compared with the antibiotic treatment group, it shows similar growth-promoting effects and can also increase the levels of IgM, IL-10, and TGF-β in serum, as well as the levels of SCFAs in the colon, indicating its positive impact on gut health (Wang et al., 2019).

Commensal bacteria produce beneficial metabolites through their metabolic activities, which can regulate gut mucosal immunity and exert anti-inflammatory effects. For example, *B. Licheniformis HD173* increases the number of Butyrate-producing bacteria, promoting Butyrate production. Butyrate, as a short-chain fatty acid, has been proven to enhance the barrier function of intestinal epithelial cells and improve gut barrier function by binding to the GPR40 receptor (Miyamoto et al., 2015). In addition, it can exert anti-inflammatory effects, such as *L. plantarum PFM105*, which increases the level of IL-10 in serum, reducing the production of pro-inflammatory cytokines, thus playing its anti-inflammatory role. IL-10 is an anti-inflammatory cytokine that can inhibit excessive immune responses and maintain immune homeostasis (Pang et al., 2022). Commensal bacteria can also inhibit the colonization and growth of pathogenic microorganisms through competitive exclusion mechanisms. For instance, *Lactobacillus plantarum PFM 105* can significantly reduce the abundance of harmful bacteria (such as *Campylobacter* and *Escherichia*) in the gut, thereby reducing the risk of viral infections (Wang et al., 2019). Commensal bacteria can enhance the integrity of the gut barrier and reduce intestinal permeability, thus preventing the invasion of pathogens and viruses. Studies have shown that Fecal Microbiota Transplantation (FMT) can increase the level of autophagy in intestinal epithelial cells and enhance gut barrier function (Cheng et al., 2018). By altering the structure of the gut microbiota, commensal bacteria promote the growth of beneficial bacteria and inhibit the proliferation of harmful bacteria, thereby enhancing the host’s antiviral capabilities. For example, supplementation with *Lactobacillus plantarum* can increase the relative abundance of beneficial bacteria in the gut and improve microbial diversity (Wang et al., 2019). Commensal bacteria play a role in antiviral effects through multiple mechanisms, including regulating immune responses, inhibiting pathogen colonization, improving gut barrier function, regulating microbial community composition, and enhancing antioxidant capacity.

Studies have shown that *Lactobacillus rhamnosus* can significantly improve the gut health of piglets infected with PEDV (Xu et al., 2024). Specifically, this is manifested as reduced intestinal pathological damage, increased villus height, and decreased crypt depth. Decreased intestinal permeability, with reduced levels of diamine oxidase and D-lactic acid. Optimized gut microbiota community structure, with increased beneficial bacteria and decreased harmful bacteria. And enhanced intestinal immune function, with increased levels of immunoglobulin IgA and improved balance of pro-inflammatory and anti-inflammatory cytokines. These results indicate that *Lactobacillus rhamnosus* alleviates intestinal damage caused by PEDV infection through multiple mechanisms, including improving intestinal barrier function, optimizing microbial community structure, and enhancing immune function.

Another study has demonstrated that the metabolites of *Lactiplantibacillus plantarum* can inhibit the proliferation of PEDV in small intestinal epithelial cells (Kan et al., 2023). The mechanism may involve regulating intracellular calcium ion (Ca^2+^) concentration and maintaining the balance of calcium ions inside and outside the cell. This regulatory effect may help maintain normal cellular physiological functions and enhance cellular resistance to viruses. During PEDV infection, the gut microbiota becomes disrupted, with a decrease in beneficial bacteria (such as *Bacteroides*, *Butyricimonas*, and *Psychrobacter*) and an increase in harmful bacteria (such as *Enterococcus*, *Fusobacterium*, *Escherichia coli*, and *Desulfovibrionaceae*). Probiotic supplementation can restore the balance of the gut microbiota and enhance intestinal barrier function, thereby mitigating damage caused by PEDV infection. Some probiotics can also compete with PEDV for binding sites, thereby preventing the virus from adhering to host cells. For example, *Limosilactobacillus reuteri* can hinder viral entry into host cells by directly interacting with the virus (Javanshir et al., 2025). This competitive mechanism can effectively reduce PEDV colonization and replication in the gut.

Probiotics can secrete a variety of metabolites with antiviral effects, such as Exopolysaccharides (EPS) and SCFAs. For example, the EPS secreted by *Lactiplantibacillus plantarum* can adhere to PEDV, preventing it from binding to host cells, and induce early apoptosis of damaged cells (Chen S, et al., 2023). Additionally, probiotics can maintain mucosal barriers by enhancing the function of tight junction proteins between intestinal epithelial cells, thereby reducing intestinal damage caused by PEDV infection. For example, *Lacticaseibacillus rhamnosus* can enhance intestinal cell barrier function by regulating the IRF3 and NF-κB signaling pathways, thereby alleviating diarrhea caused by rotavirus infection. A similar mechanism may also apply to PEDV infection (Olímpio et al., 2023). These studies indicate that probiotics can have a positive impact on PEDV infection through multiple mechanisms, including maintaining intestinal barrier function, modulating immune responses, secreting antiviral metabolites, and competing for binding sites. These findings provide a scientific basis for the potential application of probiotics in the prevention and treatment of PEDV infection.

## 3 Traditional Chinese medicine regulates the microorganisms in the intestinal tract of piglets

### 3.1 Regulation of swine intestinal microbiota and prevention of porcine epidemic diarrhea by TCM formulas

Veterinary TCM contains a variety of active ingredients, such as polysaccharides, alkaloids, tannins and phenolic acids and other compounds. Veterinary traditional Chinese medicine not only maintains and promotes the animal’s innate immune function, but also works together with the intestinal flora to effectively reduce the rate of disease in animals (**Table 1**).

**Table 1:** Chinese veterinary drugs for regulating gastrointestinal flora in piglets.

Chinese traditional medicine		Major constituent	Flavor and meridian tropism	Reference
Xia sang ju		Rosmarinic acid,Luteolin,Rutin ,and Quercetin	Cold in nature; entering the Liver meridian.	(Li et al., 2021)
Lotus seeds		Lotus seeds contain phytosterols, lactams, and other bioactive compounds.	Neutral in nature; associated with the Spleen, Kidney, and Heart meridians.	(Liu et al., 2019)
coix seed			Cold in nature; associated with the spleen, stomach, and lung meridians.	
Ge gen Qin lian Tang	Radix Puerariae	Daidzin,genistin	Cool in nature; associated with the Spleen and Stomach meridians.	(Xiao et al., 2023)
	Glycyrrhiza preparata	Glycyrrhizin,liquiritin	Neutral in nature; Associated with the Heart, Lung, Spleen, and Stomach meridians.	
	Scutellaria baicalensis	baicalin	Cold in nature; associated with the Lung, Gallbladder, Spleen, Large Intestine, and Small Intestine meridians.	
	Coptis chinensis	Berberine,coptisine	Cold in nature; associated with the Heart, Spleen, Stomach, Liver, Gallbladder, and Large Intestine meridians.	
Qi Weng Huang Bo Powder	Astragalus	Astragalus polysaccharides, Anemone saponins, and Berberine Hydrochloride	Slightly warm in nature; Associated with the Lung and Spleen meridians.	(Pi et al., 2023)
	Phellodendron		Cold in nature; Associated with the Kidney, Bladder, and Large Intestine meridians.	
	Pulsatilla chinensis		Cold in nature; Associated with the Liver, Stomach, and Large Intestine meridians.	
secondary bile acids		Lithocholic acid, Deoxycholic acid	animal-sourced traditional Chinese medicine, not yet assigned to meridian	(Dong et al., 2021)

TCM prescription has an important role in the prevention and treatment of PEDV infection by regulating the intestinal flora. Its mechanism mainly includes regulating the composition of intestinal flora, increasing the number of beneficial bacteria such as Lactobacillus and Bifidobacterium, and inhibiting the proliferation of harmful bacteria, thus reducing the production of harmful metabolites and decreasing intestinal inflammation; at the same time, the TCM can enhance the intestinal barrier function, protect the structure of intestinal mucosa, improve the dysfunction of intestines, and reduce the oxidative damage; in addition, the TCM can enhance the immune. The TCM can boost the intestinal mucosal immune response. It achieves this by modulating the gut microbiota. Additionally, it regulates the immune signaling pathway. It also inhibits the replication of porcine epidemic diarrhea virus (PEDV). Finally, it helps reduce intestinal pathological damage. For example, Baitouweng San has been shown to significantly improve the intestinal flora composition in PEDV-infected piglets. It increases the number of beneficial bacteria and inhibits the proliferation of harmful bacteria. This helps reduce intestinal damage and enhances the intestinal barrier function (Qu et al., 2025). Lizhong Tang can inhibit the replication of PEDV by regulating the intestinal flora, and promote the growth of piglets (Chen et al., 2024). Although studies have confirmed the efficacy of TCM formulas in the prevention and treatment of PEDV infection, further clarification is needed on the specific changes at the level of bacterial species and the mechanism of action, especially the mechanism of action of beneficial bacteria such as lactic acid flora.

In terms of intestinal microecology, Chinese medicine compounding provides a more favorable environment for probiotics to survive by regulating the composition of intestinal flora. For example, coix seed and lotus seed compound can regulate intestinal pH and promote the proliferation of beneficial bacteria such as *Lactobacilli* and *Bifidobacteria*, while inhibiting the growth of harmful bacteria (Li et al., 2021). This improvement in microflora balance helps to enhance the intestinal barrier function and reduce the chance of invasion by pathogens. In addition, the extracts from TCM enhance the production of SCFAs (Pi et al., 2023; Xiao et al., 2023). This improvement in microflora balance helps to enhance the intestinal barrier function and reduce the chance of invasion by pathogens. In addition, TCM enhance the production of SCFAs such as butyric acid and propionic acid, by increasing the number of butyric acid-producing bacteria, metabolites that play an important role in maintaining intestinal barrier integrity and anti-inflammation. For example, Astragalus Weng Huangbai San elevates the expression of anti-inflammatory factor IL-10 and inhibits pro-inflammatory factor TNF-α by modulating immune signaling pathways, thereby achieving immune homeostasis (Xiao et al., 2023). In terms of antioxidant, the active ingredients in the TCM, such as minuscic acid and lignans in the residue of Xia Sang Ju, can significantly increase the activity of glutathione and SOD, reduce the damage of oxidative stress on intestinal cells, and thus protect the integrity of the intestinal mucosa (Li et al., 2021). Its active ingredients, such as Minuscic acid and Lignans, can also directly inhibit the adsorption, entry, replication or assembly of PEDV. At the same time, these complexes can also promote the development of intestinal villi and enhance the expression of tight junction proteins, thus improving the integrity of the intestinal barrier and reducing intestinal permeability.Studies have shown that the addition of 1% - 2% of TCM (e.g., Xia Sang Ju residue) to diets can effectively reduce the incidence of diarrhea in piglets and improve intestinal health and antiviral resistance (Li et al., 2021). By regulating the composition of the intestinal flora, enhancing the intestinal barrier function and immunomodulatory effects, the TCM are of great value in the prevention and control of PEDV infections.

### 3.2 Single prescription of traditional Chinese medicine regulates intestinal microorganisms in piglets and prevents PED

TCM monopreparations have demonstrated significant potential in the prevention and treatment of PEDV infection through multiple mechanisms that regulate theintestinal microbiota of piglets. These TCM components can not only directly inhibit the replication and infection of PEDV but also enhance intestinal barrier function and improve the host immune response by modulating the composition and function of the gut microbiota (**Table 2**).

**Table 2:** Mechanism of anti-PED action of single traditional Chinese medicine.

Traditional Chinese medicine	Mechanism of antiviral action	Concentration/(mg/mL)	References
Dandelion	Inhibits polymerase activity and inhibits viral replication by reducing viral nucleoprotein (NP) RNA levels	3.91	(Gan et al., 2024; He et al., 2011)
Isatidis Radix	The alkaloid epigallocatechin, contained in Panax quinquefolium, signals antivirally through mitochondria in order to reduce viral susceptibility	1.95	(Gan et al., 2024)
Alisma plantago-aquatica L.	Zedoary contains triacetate derivatives that can limit the secretion of viral surface antigens and is used in the treatment of coronavirus infections and diarrhea	1.95	(Gan et al., 2024)
Puerariae Lobatae Radix	Downregulation of tumor necrosis factor expression and modulation of Th1/Th2 immune balance to reduce inflammatory response	0.98	(Gan et al., 2024; Geng et al., 2019)
Scutellariae Radix	Scutellaria baicalensis contains flavonoids that can interfere with viral replication, translation, and multiprotein processing by preventing viruses from entering cells.	0.98	(Gan et al., 2024)
Rhubarb	Blocking PEDV adsorption to cells, inhibiting virus synthesis and replication, and directly inactivating the virus	0.113~1.640	(Yuan et al., 2022; Chen et al., 2015)
Aloe	Reduces mRNA and protein levels	16	(Zhang et al., 2024; Xu et al., 2020)
Portulacae Herba	Downregulates PEDV S protein expression in a dose-dependent manner	25	(Shao et al., 2024; Liu et al., 2021)

These TCM components work together through multiple mechanisms to regulate the gut microbiota and improve intestinal health: increasing the number of beneficial bacteria by providing prebiotics or directly promoting their growth, thereby enhancing intestinal barrier function; inhibiting harmful bacteria through antimicrobial effects to reduce their growth and lower intestinal inflammation; modulating the intestinal microecology by optimizing microbial composition, improving the intestinal microenvironment, and enhancing the host immune response; and increasing antioxidant capacity by reducing oxidative stress damage, protecting the intestinal mucosa, and enhancing intestinal function. These integrated mechanisms enable these TCM components to not only prevent and treat PEDV infection but also significantly improve the intestinal health of piglets. For example, the flavonoids and alkaloids in Pueraria root (Ge Gen) can regulate the Th1/Th2 immune balance and reduce inflammatory responses. The flavonoids in Scutellaria root (Huang Qin) can prevent viral entry into cells and interfere with viral replication and translation. The alkaloids and flavonoids in Rheum palmatum (Da Huang) can block PEDV adsorption to cells and inhibit viral synthesis and replication. Additionally, the polysaccharides and flavonoids in Portulaca oleracea (Ma Chi Xian) can dose-dependently downregulate the expression of PEDV S protein, thereby inhibiting viral adsorption and entry.

These TCM monopreparations provide an effective strategy for the prevention and treatment of porcine epidemic diarrhea through multiple mechanisms, including direct antiviral effects, gut microbiota modulation, and enhancement of the host immune response, and they hold significant application value.

### 3.3 Prevention of PED by active ingredients of traditional Chinese medicine

Flavonoids, polysaccharides, terpenes, phenolic acids and ellagic acid are five major groups of natural products that synergistically inhibit PEDV infection and protect the piglet intestine through multi-targets and multi-pathways. Flavonoids (quercetin, hawthorn flavonoids, naringenin, etc.) can block viral 3CLpro/PLP2 activity, reduce ORF3 and N protein expression, and inhibit pro-inflammatory signals such as NF-κB, AP-1, and COX-2/LOX/iNOS, which has both anti-inflammatory and anti-viral effects. Polysaccharides (Astragalus, Patchouli, Amaranthus, etc.) down-regulate cytokines such as IL-1β, IL-6, and TNF-α via the TLR4/MAPK/NF-κB axis to inhibit viral invasion and replication, and at the same time, enhance IL-6 and sIgA to strengthen mucosal immunity. Terpenoids (glycyrrhetinic acid, oleanane triterpenes) inhibit viral invasion, replication, and assembly by reducing ORF3 and structural protein (S, M, N) RNA levels, and down-regulate pro-inflammatory cytokine expression.Phenolic acids (octyl gallate OG directly bind to PEDV 3CLpro, blocking viral polyprotein processing, and exerting broad-spectrum inhibitory effects on porcine coronaviruses such as PEDV, TGEV, SADS-CoV, and PDCoV.Ellagic acid (EGCG, proanthocyanidins) prevented viral attachment and entry by decreasing ORF3 mRNA and N protein expression, and inactivated viruses in a dose-dependent manner.

In addition, these natural substances indirectly stimulate the immune response of the host, thus effectively neutralizing the PEDV virus (**Table 3**). Natural compounds such as Flavonoids, polysaccharides, alkaloids, and terpenoids have been shown to inhibit the expression of PEDV structural and nonstructural protein genes to varying degrees. Particularly, since PEDV mainly attacks the epithelial cells of porcine small intestine, leading to the damage of the small intestine and mucosal detachment, the herbal medicine can significantly improve the morphology and structure of the intestinal tract.In addition, Chinese medicines can increase the thickness of the intestinal mucosa, improve the ratio of the height of villi to the depth of crypts, and result in a neater and more homogeneous structure of the striated rim of the intestinal mucosal surface and a clearer structure of the lamina propria (Bouvier et al., 2005). Active ingredients such as polysaccharides can also improve the intestinal microbial environment, which can have a therapeutic effect on PED.In addition to directly affecting the intestinal structure and internal environment, herbal medicines can also enhance the overall immunity of infected pigs. A variety of Chinese medicines and their formulas effectively enhance the innate immunity of the organism against PEDV infection by promoting the humoral and cellular immune functions of the organism and increasing the secretory antibody secretion and T-lymphocyte proliferation in the intestinal tract.

**Table 3:** Anti-pedv mechanism of active components of traditional Chinese medicine.

Classification	Antiviral active ingredient	Antiviral effect	References
Flavonoids	Quercetin 7-rhamnoside	Inhibits viral replication at early infection stage	Li et al., 2020
	Quercetin	Inhibits enzyme activity by binding to 3CLpro	Li et al., 2020
	Hawthorn total flavonoids	Reduces ORF3 gene expression; reduces N protein RNA expression levels	Sun H. B et al., 2024
	Naringenin	Binds to 3CLpro protein or PLP2 protein and inhibits enzyme activity	Gong et al., 2023
Polysaccharides	Patchouli polysaccharide	Inhibits PEDV replication; inhibits PEDV penetration and replication	Chen et al., 2020
	Amaranthus polysaccharide	Reduces inflammatory cytokine expression; inhibits NF-kappaB signaling pathway; inhibits PEDV replication	Liu et al., 2021
Terpenoids	Glycyrrhizic acid	Reduces inflammatory cytokine expression; affects PEDV invasion; inhibits PEDV replication	Huan et al., 2017
	Oleanane	Reduces N-protein, S-protein, and M-protein RNA expression levels of PEDV	Yang et al., 2015
Phenolic acids	Gallic acid	Inhibits enzyme activity by binding to 3CLpro; inhibits PEDV invasion	Su et al., 2023
Tannins	(-)-Epigallocatechin gallate	Reduces ORF3 mRNA expression level; inhibits N protein RNA expression	Huan et al., 2021

## 4 Conclusions and future perspectives

Porcine epidemic diarrhea virus (PEDV) infection significantly affects the composition and function of the gut microbiota of piglets. The intestinal microorganisms of piglets are important for the maintenance of the immune barrier and the structural integrity of the intestinal tract. So it is important to regulate the intestinal microflora colonization according to the influencing factors.

It was shown that PEDV infection resulted in an imbalance in the microbiota of the ejunal contents and jejunal mucosa, with the abundance of the *Firmicutes* being higher in uninfected piglets and the abundance of the *Ascomycetes phylum* being lower in uninfected piglets, which suggests that PEDV infection has a significant impact on the composition of the intestinal microbiota of piglets (Huang et al., 2018; Wu et al., 2024). In addition, secondary bile acids sourced from the gut microbiota, such as LCA and DCA, play an important role in maintaining the microbial diversity of the intestinal tract and protecting the host from PEDV infection.

The gut microbiota is a key factor in regulating host health, which not only influences gut barrier function, but is also involved in the production of SCFAs, which are essential for gut health. PEDV infection may affect gut barrier function and host immune response by influencing the composition and function of the intestinal flora, which in turn affects the production of SCFAs (Benjamin et al., 2000). In particular, SCFAs such as Butyric acid promote the production of antiviral interferons through activation of the GPR43 receptor, thereby limiting PEDV replication (Pi et al., 2023).

The process of colonization of the intestinal flora of piglets plays a crucial role in their health, development of the immune system and growth performance. When piglets are exposed to a bacterial environment after birth, a variety of microorganisms enter the organism, consume maternal colostrum, and survive and multiply after initial colonization by microorganisms selected to use nutrients from breast milk as substrates. The diversity of microorganisms increases with the age of the host and gradually forms a stable microbiota, which plays an important role in the growth and health of the host. There are significant differences among in the composition and function of the microbiota in different parts of the intestinal tract of piglets. For example, the small intestine is dominated by *Lactobacillus* spp. while in the colon and cecum, *Anabaena* spp. and *Clostridium* spp. produce SCFAs through the fermentation of dietary fiber to provide energy to the host and regulate the immune response (Dong et al., 2021).

Chinese medicinal preparations have shown potential in regulating the intestinal microbiota and enhancing the intestinal barrier function in piglets. It has been found that CHM such as Qi-Weng-Huang-bai can effectively regulate the balance of intestinal microflora in pigs (Xiao et al., 2023). The effective active ingredients therein, such as astragalus polysaccharides, baicalin saponins, and berberine hydrochloride, enhanced the intestinal barrier function by intervening to increase the diversity of intestinal microflora and promoting the proliferation of favorable bacteria (Xiao et al., 2023). Astragalus membranous can increase intestinal microbial diversity, promote the proliferation of beneficial bacteria, and restore the composition of intestinal flora and re-establish immune homeostasis by increasing the levels of Butyric acid, Valeric acid and Isovaleric acid (Yin et al., 2024). Berberine chloride, on the other hand, maintains the dynamic balance of intestinal flora by regulating the intestinal flora, increasing the content of Volatile fatty acids (VFAs), and decreasing pH, thereby enhancing host resistance to pathogens and preventing adverse inflammatory responses (Wu et al., 2022).

The effect of PEDV on the intestinal microbiota of piglets is a complex process that involves several aspects, including the composition of the microbiota, intestinal barrier function, and host immune response. These effects interact and together determine the health status and disease course of PEDV-infected piglets. Understanding the mechanisms of these influences is essential for developing effective prevention and treatment strategies. The current study demonstrates that PEDV infection leads to significant changes in the gut microbiota of piglets, particularly in the abundance of Firmicutes and Proteobacteria. In addition, gut microbiota-derived secondary bile acids, such as LCA and DCA, play an important role in maintaining microbial diversity in the intestinal tract and protecting the host from PEDV infection (Mann et al., 2024; Xing et al., 2024). The results indicated that SCFAs-producing microflora dominated the intestinal tract of healthy piglets and were effective in suppressing PEDV-induced intestinal flora imbalance (Mann et al., 2024). SCFAs such as Butyrate promote the production of antiviral interferon by activating the GPR43 receptor, thus limiting PEDV replication (Tsui et al., 2025).

The central bottleneck in current PEDV research is the inability to distinguish whether changes in flora are drivers of infection or immune by-products, and there is an urgent need to validate the causal role of butyric acid-producing or bile acid-metabolizing bacteria on PEDV susceptibility by using a single-bacteria colonization model in a sterile piglet. At the same time, the dosage threshold for the butyric acid-GPR43-IFN pathway has not yet been quantified, and jejunum organoids can be used to determine the ≥5 mM butyric acid exposure for 24 hours induces athe minimum effective condition for a 3-fold up-regulation of IFN-β. The absence of strain-level targets for TCM active ingredients needs to be demonstrated by sterile piglet colonization and LC-MS metabolome. In addition, the spatial and temporal sequence of TCM-probiotics synergism was not clarified, and three temporal treatments, namely, “TCM first, probiotic second”, “probiotic first, TCM second” and “synchronized administration”, need to be set up in the same batch of piglets to determine the effectiveness of FMT in doubling colonic butyric acid and reducing diarrhea score. We need to set up three temporal treatments to explore the spatial and temporal sequence of drug-probiotics synergism in the same batch of piglets.In addition, the low titers of PEDV in culture and the continuous mutation of viral strains make the prevention and control of PEDV highly challenging. Moreover, there are currently no effective treatments available in clinical practice. Therefore, exploring drug targets for PEDV treatment is of great significance for its prevention and control. Another difficulty is that the gut microbiota of neonatal piglets lacks diversity, leading to immature innate intestinal immunity, which increases their susceptibility to PEDV. Future research needs to further explore the specific changes in the gut microbiota after PEDV infection and how these changes affect the health and disease resistance of piglets. In particular, it is necessary to investigate how to enhance piglets’ resistance to PEDV by modulating the gut microbiota. For example, fecal microbiota transplantation to colonize more SCFA-producing microbes in the host may prevent PEDV infection. In addition, TCM formulations provide new preventive and therapeutic strategies for modulating the gut microbiota and enhancing intestinal barrier function. These strategies lay the foundation for the development of effective preventive measures, but their specific mechanisms of action and effects still need further research and validation. In addition, the therapeutic effect of TCM is relatively slow. Compared with chemical drugs, TCM usually takes a longer time to show significant therapeutic effects. During the acute phase of PEDV infection, piglets may rapidly develop severe diarrhea, dehydration, and other symptoms. At this time, TCM may not be able to relieve symptoms in time and cannot meet the clinical needs for rapid disease control. Therefore, in practical applications, TCM often needs to be used in combination with other fast-acting drugs (such as antiviral drugs or antibiotics) to achieve the dual goals of rapid symptom relief and long-term regulation of the body’s condition. Moreover, the quality control of TCM is relatively difficult. The quality of traditional Chinese medicinal materials is affected by factors such as origin, growth environment, and harvesting time. There may be significant differences in the content and activity of components in different batches of traditional Chinese medicinal materials. In addition, the preparation process of TCM is complex, and different preparation methods may lead to changes in the structure and activity of TCM components. These factors together affect the stability and effectiveness of TCM formulations, making quality control a major bottleneck restricting the widespread application of TCM. At present, although some quality standards and testing methods for TCM have been established, compared with chemical drugs, the quality control system of TCM is still not perfect and cannot achieve precise quality control and therapeutic effect prediction.

Despite the many challenges faced in the prevention and control of PEDV using TCM, the future prospects remain very promising. With the in-depth research on PEDV infection mechanisms and gut microbiota, the unique advantages of TCM in modulating gut microbiota and enhancing immune function will be more fully realized. Recent studies have already revealed the close relationship between gut microbiota and host health. TCM can effectively improve the intestinal microenvironment and enhance the host’s immune response by modulating the composition and function of gut microbiota, thus providing new ideas and strategies for the prevention and control of PEDV. In addition, exploring the synergistic effects of TCM with other green prevention and control measures such as probiotics and prebiotics will become an important direction for building a more comprehensive green prevention and control system for PEDV. Probiotics and prebiotics can directly modulate gut microbiota and improve the intestinal microenvironment, while TCM can enhance the host’s immune function and overall health status through various pathways. Combining these green prevention and control measures can not only play to their respective strengths but also achieve synergistic effects, reducing the dependence on chemical drugs and lowering the occurrence of drug resistance, providing strong support for the sustainable development of the pig farming industry. Personalized prevention and control plans based on individual differences will also become an important development direction for the application of TCM. The composition of gut microbiota, immune status, and drug response vary among individual piglets. Therefore, future TCM prevention and control strategies will focus more on individualization. By monitoring and analyzing the gut microbiota of individual piglets and combining their health status and growth environment, personalized TCM formulations and usage plans can be developed to achieve precise prevention and control and improve the effectiveness of prevention and control.

In summary, the effects of PEDV infection on the intestinal microbiota of piglets are multifaceted, involving various aspects such as the composition of the microbiota, intestinal barrier function, and host immune response. These influences interact with each other and together determine the health status and disease course of PEDV-infected piglets. Understanding the mechanisms of these influences is essential for developing effective prevention and treatment strategies. Future studies need to further explore the specific changes in gut microbiota post PEDV infection and how these changes affect the health and disease resistance of piglets. Meanwhile, CHM preparations provide new preventive and therapeutic strategies in regulating the intestinal microbiota and enhancing the intestinal barrier function, laying the foundation for the development of effective preventive measures in the future.
